# Processing of Bulk MgB_2_ Superconductors by Coupling Laser Powder Bed Fusion and Spark Plasma Sintering Techniques

**DOI:** 10.3390/ma18102367

**Published:** 2025-05-19

**Authors:** Anastasia Sklyarova, Lionel Presmanes, Vincent Baylac, Geoffroy Chevallier, Claude Estournès, Benjamin Duployer, Jacques Noudem, Pierre Bernstein, Philippe Tailhades, Yohann Thimont

**Affiliations:** 1 Centre Inter-universitaire de Recherche et d’Ingénierie des Matériaux, CNRS, INPT, UPS, Université de Toulouse, 118 Route de Narbonne, 31062 Toulouse, Cedex 9, France; asklyaro@gmail.com (A.S.); lionel.presmanes@univ-tlse3.fr (L.P.); geoffroy.chevallier@univ-tlse3.fr (G.C.); claude.estournes@univ-tlse3.fr (C.E.); benjamin.duployer@univ-tlse3.fr (B.D.); philippe.tailhades@univ-tlse3.fr (P.T.); 2 ENSICAEN, 6, Boulevard Maréchal Juin—CS 45053, 14050 Caen, Cedex 4, France; jacques.noudem@ensicaen.fr (J.N.); pierre.bernstein@ensicaen.fr (P.B.)

**Keywords:** additive manufacturing, laser powder bed fusion, spark plasma sintering, superconductors, MgB_2_

## Abstract

This article demonstrates the concept proof to manufacture parts of MgB_2_ by Laser Powder Bed Fusion (L-PBF) coupled to Spark Plasma Sintering (SPS) by an optimization of the L-PBF and SPS conditions to limit the phase degradation and complete the sintering. Optimal L-PBF parameters were identified in order to obtain the material preforms with a minimal degradation of the MgB_2_ phase, and then these preforms were sintered by SPS using an inert powder as matrix with a purpose to receive a mechanically more reliable product. Sintered samples show superconductivity state inherent for the raw material and demonstrate superconducting transition around 38 K according to the magnetic moment measurements.

## 1. Introduction

Superconducting materials begin to conquer more and more of our daily life. Superconducting parts find its applications in medicine as MRI superconducting coils, in industry as electromagnets and magnetic shields, in transport as parts of motors and levitation transportation systems, in superconducting cavities, and in many other fields [1,2,3,4]. So, the number of potential applications of superconducting materials are constantly increasing. Thus, regarding the non-consideration of possibly discovering completely new superconductors, the main goal is to find suitable materials with appropriate properties among the already existing superconducting compounds.

Despite some difficulties in implementation, MgB_2_ is one of the promising materials for practical applications. MgB_2_ is an intermetallic compound with low density (2.5 g/cm^3^). While this compound has been known since the 1950s, its superconductivity state was only discovered in 2001. Its critical temperature is 39 K [5] and its critical current density in self-field is 10^6^ A/cm^2^ at 10 K [6]. MgB_2_ has a simple stoichiometry in comparison to the high-*T*_c_ oxide superconductors such as YBa_2_Cu_3_O_7_, whose superconducting properties strongly depend on an oxygen content [7]. Moreover, MgB_2_ contains only cheap and non-toxic elements. Therefore, MgB_2_ could be advantageously used in superconducting motors, magnets, and magnetic shields [8]. Although Spark Plasma Sintering (SPS) is successfully used for sintering MgB_2_ pellets [9], the dense fabricated pellets show high mechanical hardness of 12.5 GPa [10]; moreover, the MgB_2_ phase can be easily oxidized in air and water, which reduces the number of its practical applications because of difficulties in further processing of pellets to obtain complex shaped samples [11].

A possibility to resolve this problem is to replace current manufacturing technology by techniques resulting in samples with the wanted shapes just after sintering. Modern methods of Additive Manufacturing (AM) techniques allow to make layer-by-layer 3D parts with complex shapes [12] from a wide range of powder materials as 316L [13] and titanium metallic alloys. AM techniques are also figures as new research interests for functional materials [14] and also, more recently, high temperature superconductors [15]. From a more general point of view AM techniques generate a colossal interest for industry, research, and society [16]. Laser powder bed fusion (L-PBF) is the most commonly used AM technique due to its large scaling capability which is well suited for industrial production. Nevertheless, optimal parameters of the L-PBF such as laser power density, scanning speed, layer powder thickness, etc., depend on the materials properties and for most of them require a careful study for their practical implementation. In addition, many materials elaborated by AM techniques may require sintering post treatment to achieve satisfactory characteristics.

An innovating concept proof for producing MgB_2_ superconducting parts by combining L-PBF and SPS techniques is proposed in this paper. The development of the proposed concept of manufacturing could help to overcome some technical limitations for future challenging superconducting applications concerning the MgB_2_ material.

## 2. Materials and Methods

For manufacturing MgB_2_ superconducting parts, a commercial raw powder produced by PAVEZYUM (Gebze Kocaeli, Türkiye) was used. Purity of the raw powder was more than 95%, and the particle size was from 0.7 µm to 5 μm with some dispersed large agglomerates >10 µm (given in the next section); the granulometric distribution in number is given in the Supplementary File. Before the manufacturing process and after each synthesis step, the purity of the precursor powder as well as the phase composition of the produced samples were checked using X-ray Diffraction method (XRD) in the Bragg–Brentano configuration with the Bruker D8 diffractometer (Billerica, MA, USA). The obtained XRD patterns have been refined using the Rietveld method in FullProf program [17].

Prior to the beginning of Laser Powder Bed Fusion, the aggregates of raw MgB_2_ powder have been broken to individual particles with the Kakuhunter Mixer SK-300SII (SHASHIN KAGAKU Company, Shiga, Japan) device. The following conditions were used for the Mixer work: the rotation speed was 1100 rpm, and the working time was 6 min for a maximum mass of 180 g.

In the next step, the prepared powder was introduced into a chamber of the L-PBF Phenix ProXLS 200 (Rock Hill, SC, USA) apparatus with an inert argon atmosphere containing less than 800 ppm of oxygen with the purpose to obtain preformed samples.

After subsequent studies like XRD, porosity checking, and microscopy, the obtained preforms underwent sintering using the Spark Plasma Sintering technique with the Sumitomo Dr. Sinter 2080 (Tokyo, Japan) apparatus, available at the Plateforme Nationale de Frittage Flash in Toulouse (France). The sintering step was carried out in an Ar (4.5) atmosphere using an inert sacrificial matrix powder and was used to obtain the denser parts. The final dense samples were characterized by X-ray Diffraction (Bruker D8), as well as by Scanning Electron Microscopy (SEM) using the TESCAN VEGA 3 (Kohoutovice, Czech Republic) microscope in a high vacuum mode with 20 kV accelerating voltage.

Superconductivity properties of selected samples were investigated by magnetic susceptibility measurements using a SQUID magnetometer, Quantum design MPMS 5, (San Diego, CA, USA) in the temperature interval of 20–40 K in both the zero-field cooling (ZFC) and the field-cooling (FC) modes.

## 3. Results

The Laser Powder Bed Fusion method is based on the interaction between a high-energy laser beam and a raw powder. At the first steps of the work with a new material experimental determination of L-PBF parameters is extremely important because unwanted phases may arise and grow up in the material during the fusion leading to disappearing of some properties important for practical application. Because of the absence of any information about MgB_2_ superconducting parts produced by the L-PBF method, a huge part of this study was devoted to seek the optimal L-PBF conditions. For this purpose, the simple cuboid shape with the geometry sizes of 1 × 1 × 0.4 cm^3^ was chosen for preformed samples and the following synthesis conditions have been experimentally tested in a wide range: the power of laser (*P*) {30; 130} W with the laser spot diameter of 1 mm, the laser scanning rate (v) {50; 800} mm/s and the hatching space (Δ) {100; 250} μm. Based on the sample dimensions, 64 samples fused at different L-PBF conditions were possible to obtain each run but, in the future, the size of the manufactured part may be limited to the size of the Job box of the L-PBF apparatus (14 × 14 × 10 cm^3^ in our case).

Layer thickness is one of the critical parameters for L-PBF. Testing the different powder bed thickness values in the range from 50 to 300 μm at various values of laser power has shown that for the low thickness (50 μm), the MgB_2_ phase degradation becomes too high due to overheating of the MgB_2_ powder bed. At 100 μm and above, the result showed the highest number of good-synthesized and hard enough preforms. However, for bed thicknesses higher than 100 μm, the preforms were brittle due to insufficient sintering between each layer. Thus, among all tested conditions, at the powder bed thickness of 100 μm, the L-PBF fabrication yield (viable samples) was the highest and amounted to ∼85%.

**Figure 1:**
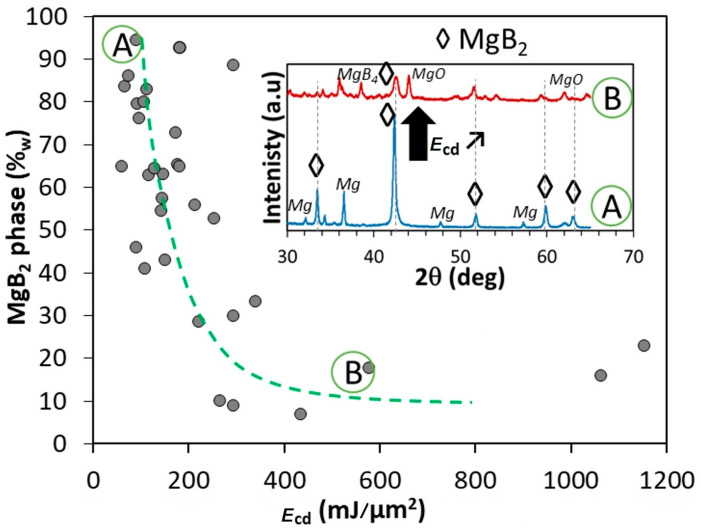
MgB_2_ phase weight fraction versus *E*_cd_, in inset: XRD patterns as example of phase degradation evolution with increasing *E*_cd_. A and B correspond to two samples made with two different *E*_cd_. The dashed curve is a guide line for the eyes.

The cumulative energy dose (*E*_cd_), which was received by the raw powder on working area (working plate), was defined by the cumulated energy given by 3D Gaussian distribution on 1 μm surface area. Influence of *E*_cd_ on the value of MgB_2_ weight fraction is presented in Figure 1.

As seen in the figure, the L-PBF process in many cases triggers the formation of impurity phases (Mg, MgO, MgB_4_, etc.), which leads to the reduction in the MgB_2_ fraction and to the possible generation of unwanted properties in the obtained preforms. Two samples marked as A and B, which were achieved using *E*_cd_ = 89 mJ/μm^2^ and *E*_cd_ = 576 mJ/μm^2^, respectively, are shown in the figure. The Figure 1 inset shows the comparison of XRD patterns of these samples. A conclusion about the degradation of the main MgB_2_ phase with increasing *E*_cd_ can be drawn from this picture.

The highest value of the MgB_2_ weight fraction was found for *E*_cd_, included in the 60–200 mJ/μm^2^ domain. Below 60 mJ/μm^2^, the sintered layers crumbled to powder, and above ∼200 mJ/μm^2^, the fraction of the MgB_2_ phase in the obtained samples rapidly decreased (Figure 1 insert), while the samples themselves were overheated.

**Figure 2:**
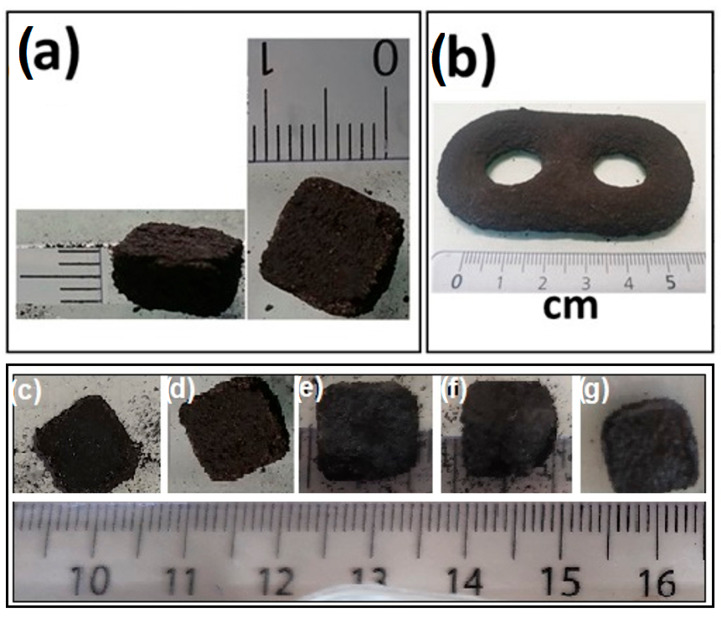
Pictures of the fabricated MgB_2_ parts: (**a**) Picture of the cuboid preform; (**b**) Example of preform with a complex shape elaborated in similar conditions; (**c**–**g**) Pictures of the cuboid-shaped samples after L-PBF manufactured at the different values of *E*_c__d_: (**c**) 59 mJ/μm^2^; (**d**) 89 mJ/μm^2^; (**e**) 111 mJ/μm^2^; (**f**) 212 mJ/μm^2^; (**g**) 576 mJ/μm^2^.

Even if L-PBF has been carried out within the optimal domain of *E*_cd_, the obtained samples remained brittle and showed the porosity of ∼60% as deduced from their geometric measurements and weight. These results indicate that the energy of the laser beam is not large enough for achieving a full sintering, but it is sufficient for making the preformed samples with the desired shapes like the cuboid or, for example, the double ring reported in Figure 2a and Figure 2b, respectively.

The influence of the L-PBF parameters used on the perfection of obtaining a cuboid shape of the samples is shown in Figure 2c–g. As seen in the figure, no significant effect of the *E*_cd_ value on the shape of the obtained samples is seen, and the deviation of the cuboid shape can be explained by the porosity and, consequently, the brittleness of the preforms with respect to hand manipulation.

**Figure 3:**
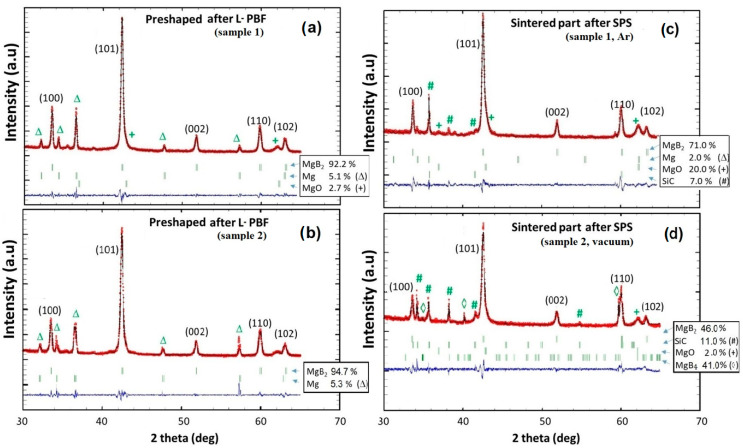
XRD patterns of the preform with 92% of MgB_2_ (**a**) and the final part (the same preform) obtained after SPS (**c**) (sample 1), and XRD patterns for comparative sample with 95% of MgB_2_ after SLS (**b**) and SPS (**d**) (sample 2). The red lines correspond to the Rietveld refinement patterns, the blue lines are the difference between the Rietveld and the experimental patterns.

After careful investigations, a preform containing more than 92% of MgB_2_ was obtained with the following L-PBF parameters: *P* = 66 W, v = 800 mm/s and Δ = 100 μm. The cumulative energy dose value was *E*_cd_ = 89 mJ/μm^2^. The XRD pattern of this sample, designated as sample 1, is presented in Figure 3a. Despite the high content of the MgB_2_ phase, weak amounts of free Mg (5%) and MgO (3%) were identified due to partial decomposition of the MgB_2_ phase and oxidation, respectively. The thorough XRD study of received samples has shown that the preforms manufactured with similar *E*_cd_ show similar XRD patterns.

For solving the problem of porosity, a specific SPS additional treatment adapted from the one developed to generate complex 3D shapes directly by SPS [18,19] was applied to cuboid samples obtained using L-PBF. The optimal preform was placed into 20 mm diameter graphite die, embedded in a sacrificial matrix powder and heated up to 800 °C under the pressure of 0.3 MPa for 8 min. Subsequently, a uniaxial pressure of 50 MPa was applied for 20 min at the temperature of 800 °C. Then, the pressure was released, and the sample was naturally cooled before to be extracted from the SPS die. The value of temperature for ex situ SPS synthesis has been chosen after testing various temperatures in a wide region from 750 °C to 1100 °C and 800 °C showed the best results with the MgB_2_ wt% vs. superconducting properties ratio point of view. Although some reports show good ex situ SPS results at higher temperatures, there are a number of works reporting the problem of MgB_2_ phase decomposition with increasing sintering process temperature [20,21], which are consistent with our results.

The choice of matrix for the SPS densification was carried out based on the analysis of the literature data and available materials. Several types of sacrificial matrix powder such as Al_2_O_3_, MgAl_2_O_4_ and BN have been tested for the SPS process. The two first matrix (Al_2_O_3_ and MgAl_2_O_4_) caused the phenomenon of interdiffusion and oxidation, which strongly increase the presence of undesirable phases in the final sample as MgO (>40%_w_) while BN powder can unbalance the ratio MgB_2_/MgB_4_ in favour of the last one. Based on the literature, it was expected that the BN matrix would give the best result in terms of preserving the initial phase of MgB_2_, since the melting point of BN is about 3000 °C. However, the use of BN powder leads to the presence of impurities and increases the MgB_2_ phase degradation. This result is confirmed by works in which the reaction between MgB_2_ and BN was also observed. These undesirable chemical reactions have been explained by unconventional activation effects due to the pulsed current heating the sample [22,23]. Only commercial SiC powder (Sigma ALDRICH, St. Louis, MO, USA; 200 mesh particles size) made it possible to maintain a high content of the MgB_2_ phase without mutual diffusion and/or reaction. Although SiC appears to be reactive even at low temperatures, the good results obtained do not contradict previous data. It was previously noted that although SiC nanopowder reacts with MgB_2_, micro-SiC may not form any impurity phase and even nano-SiC does not always react with MgB_2_, since the possibility of reaction depends on the temperature and time of synthesis, as well as on the crystallinity of SiC [24,25,26].

The XRD pattern of sample 1 sintered using SPS in an Ar atmosphere with SiC as the sacrificial matrix is shown in Figure 3c. The resulting sample contains mainly MgB_2_ phase (71%) with a trace of Mg (2%) and with MgO content increased to 20%. A small amount of SiC on the X-ray Diffraction pattern is due to inclusions of the matrix powder on the sample surface.

The XRD patterns illustrating how the SPS conditions influence the final result are shown in Figure 3c,d. Although sample 2 after SLS synthesis showed a good MgB_2_ phase volume fraction similar to sample 1 (see Figure 3b), changing the SPS atmosphere from argon to a vacuum leads to a decrease in the MgB_2_ phase fraction. A vacuum atmosphere is unfavorable due to the decomposition of MgB_2_ into MgB_4_ according to the Le Chatelier thermodynamic principia due to the low partial pressure of vaporisation of the magnesium (MgB_2_ → MgB_4_ + Mg) [27]. The best solution to prevent decomposition is to use an inert gas such as Ar, which limit the loss of Mg and, thus, the decomposition of the MgB_2_ into MgB_4_ without the unwanted chemical reaction as with nitrogen [28].

**Figure 4:**
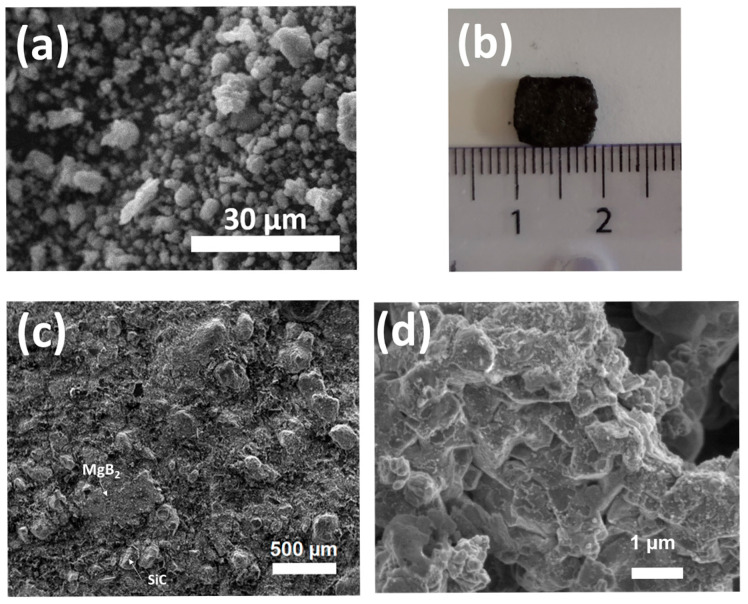
(**a**) SEM of the powder; (**b**) Top view of the sintered cuboid preform; (**c**) SEM micrograph of its surface; (**d**) SEM micrograph of the sample at higher magnification (sample 1).

The resulting cuboid parts showed undistorted square (Figure 4b), along with shrinkage of their thickness by a factor of ∼2/3 along the pressure axis, which is in agreement with a densification mechanism. This shrinkage leads to a final porosity rate of ∼15%. As a SEM micrograph shows (Figure 4c), the surface remains rough due to the SiC particles inclusion, but no porosity is visible after the sintering processing. An SEM image at high magnification to focus on the MgB_2_ microstructure is shown (Figure 4d). It is possible to identify some lamellar stacked interconnected grains resulting in the typical microstructure of the sintered MgB_2_ (resulting of its hexagonal structure). The grains of the sample are interconnected sheets, and the global melted domains have an approximate size of some micrometers (in the plane). The interconnexions between sheets proves an effective sintering mechanism, which is necessary for superconducting properties.

**Figure 5:**
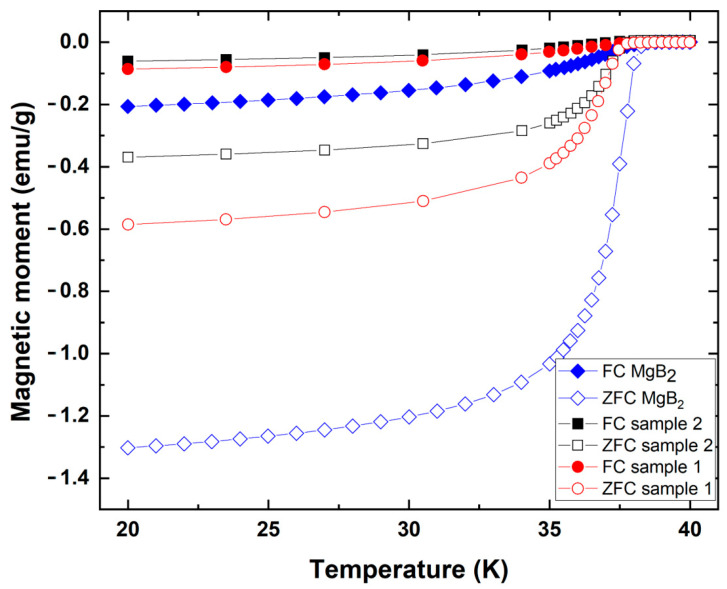
Magnetic moment versus temperature curves of the raw MgB_2_ powder (opened and closed diamond marks) and the sintered cuboid part after the final SPS step marked as sample 1 on the picture (opened and closed circles), and the comparative sample 2 after the final SPS step (opened and closed squares). FC-mode measurements have been performed under a 20 Oe applied magnetic field.

Magnetic susceptibility measurements have been carried out in the ZFC and FC regimes under a 20 Oe applied field on both samples (see Figure 5).

The magnetic susceptibility versus temperature curves for both the commercial raw powder and the sintered samples are relatively broad, and while the onset of the superconducting transition occurs at *T*_c_ equals 38 K for the raw powder, it occurs at a slightly lower temperature for the cuboid. Thus, the used two-stage (L-PBF + SPS) technology for manufacturing parts does not remarkably change the value of the critical temperature compared to the original commercial MgB_2_ powder. As could be expected, the magnetic moment of the FC samples are much smaller than those of the ZFC ones. The reason is that after ZFC, the magnetic field in the samples must be kept equal to zero if the applied field is smaller than *H*_c1_. Large magnetic moments result from the large shielding currents required for this purpose. The magnetic field is not suppressed in the superconductors after Field Cooling but canalized along vortex lines. As a result, the generated shielding currents and magnetic moments are much smaller. In addition, it is observed that at low temperature, after ZFC, the magnetic moment of the powder shows a larger amplitude than that of the sintered sample. The probable reason is the reduction in the MgB_2_ content in the sintered sample due to the large amount of MgO. As a result, one can expect a reduction in its critical current density.

The ZFC and FC magnetic susceptibility measurements were carried on sample 2 for comparison. This sample showed a higher degradation of the MgB_2_ phase after the final SPS step and very low superconducting properties with a lower magnetic moment (Figure 3d). The decrease in the superconducting properties is explained by the reduction in the MgB_2_ content. Figure 5 shows that neither the powder nor the sintered samples are completely superconducting well below the onset of superconductivity. In both cases, it is probably due to the poor grain connectivity, expected in the powder and resulting from the high amount of MgO in the sintered samples. As a consequence, the critical current density of the samples is necessarily very low. Future works should aim to decrease the MgO content in the used powder in order to obtain a narrow transition width and an appreciable critical current density and higher magnetic moments. Some authors reported a value of 2.4 emu/g for pure MgB_2_ powder, which is relatively close to our value for the tested powder [29]. Other authors such as D.H.L. Ng and W.M. Hon, in the case of the MgB_x_ with x = 2, obtain a magnetic moment of 11 emu/g at 20 K instead of 1.3 in our case [30]. It shows that the properties of the initial powder can have broad distribution in terms of semiconducting properties according to the purity and grain size. It is also known that the addition of specific elements such as Ag increases the links between the grains of MgB_2_, which enhances the superconducting properties and could be tested in future works using this process to increase the superconducting properties of the final parts [31].

The results obtained in the manufacture of formed superconducting parts according to the developed method show promising solution to make parts of MgB_2_ with specific shapes for various future superconducting applications such as magnetic shields, motors, and superconducting cavities.

## 4. Conclusions

This work describes the innovative concept of the L-PBF additive manufacturing of 3D MgB_2_ preforms using a commercial MgB_2_ powder. The cumulative energy dose of 89 mJ/μm^2^ makes it possible to obtain preforms with a practically non-degraded MgB_2_ phase. The solution to the problem of brittleness and porosity was achieved by using the SPS step (800 °C, 50 MPa, using SiC matrix powder) in argon atmosphere as a finishing treatment. The best sample shows a superconducting transition at ~38 K. These results are encouraging because they maybe provide a new opportunity to develop the intricately shaped undegraded MgB_2_ superconducting parts required for a new type of superconducting devices for various future applications concerning this superconducting material as the motors for the electric planes, the Maglev rails, coils, superconducting cavities, stetarators, etc.

## 5. Patents

This research has been patented as reference: WO2023232787 (A1) entitled Fabrication de pièces de formes complexes de céramique MgB_2_ par fabrication additive laser, Y. Thimont, L. Presmanes, A. Sklyarova, (2023).

## Data Availability

The original contributions presented in this study are included in the article/Supplementary Material. Further inquiries can be directed to the corresponding author.

## Supplementary Materials

The following supporting information can be downloaded at: https://www.mdpi.com/article/10.3390/ma18102367/s1, Figure S1: Granulometric distribution of the MgB_2_ powder.
