# Supplementary material for: Processing of Bulk MgB2 Superconductors by Coupling Laser Powder Bed Fusion and Spark Plasma Sintering Techniques
Source: Materials (Basel). 2025 May 19;18(10):2367. doi: 10.3390/ma18102367 (PMC12113012; doi:10.3390/ma18102367)
Supplement: Supplementary file 1 [file materials-18-02367-s001.zip › materials-3615746-supplementary.pdf]

Supplementary information

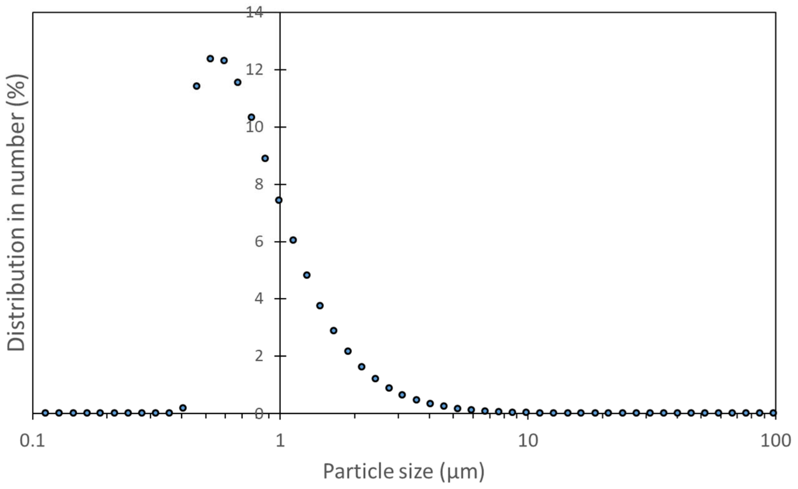

Supplementary Figure S1. Granulometric distribution of MgB_2 powder used in the study.
